# Hybrid spatial Gillespie and particle tracking simulation

**DOI:** 10.1093/bioinformatics/bts384

**Published:** 2012-09-03

**Authors:** Michael Klann, Arnab Ganguly, Heinz Koeppl

**Affiliations:** ^1^ BISON Group, Automatic Control Lab, ETH Zurich, Switzerland

## Abstract

**Motivation:** Cellular signal transduction involves spatial–temporal dynamics and often stochastic effects due to the low particle abundance of some molecular species. Others can, however, be of high abundances. Such a system can be simulated either with the spatial Gillespie/Stochastic Simulation Algorithm (SSA) or Brownian/Smoluchowski dynamics if space and stochasticity are important. To combine the accuracy of particle-based methods with the superior performance of the SSA, we suggest a hybrid simulation.

**Results:** The proposed simulation allows an interactive or automated switching for regions or species of interest in the cell. Especially we see an application if for instance receptor clustering at the membrane is modeled in detail and the transport through the cytoplasm is included as well. The results show the increase in performance of the overall simulation, and the limits of the approach if crowding is included. Future work will include the development of a GUI to improve control of the simulation.

**Availability of Implementation:** www.bison.ethz.ch/research/spatial_simulations.

**Contact:** mklann@ee.ethz.ch or koeppl@ethz.ch

**Supplementary/Information:** Supplementary data are available at *Bioinformatics* online.

## 1 INTRODUCTION

Existing methods to model the spatial–temporal dynamics in signal transduction can be grouped into (i) partial differential equation (PDE) methods (Kholodenko, 2006; Slepchenko *et al.*, 2003); (ii) spatially resolved Markovian population models, often simulated with the SSA/Gillespie method (Elf *et al.*, 2003; Gillespie, 1976; Stundzia and Lumsden, 1996) or other lattice-based methods (Angermann *et al.*, 2012; Arjunan and Tomita, 2010) and (iii) particle-based methods such as *Smoldyn* (Andrews and Bray, 2004), Greens function reaction dynamics (*GFRD*) (van Zon and Ten Wolde, 2005) and others (Klann *et al.*, 2011a; Lipková *et al.*, 2011; Plimpton and Slepoy, 2003; Pogson *et al.*, 2006).

Naturally, more detailed methods are computationally much more demanding, which requires careful selection of the right method for the biological problem in focus. Note that particle numbers and parameters are very heterogeneous in biological systems: molecule abundances range from 1 (gene) to several thousands of proteins of each class, similarly the size of the molecules and structures ranges from atoms/ions (< 1nm) to molecular complexes (2–25nm) and further to cellular sub-compartments and cytoskeleton structures (50+nm), whereas chemical interaction rate constants cover several orders of magnitude (Alberts *et al.*, 2002). Hybrid methods can be employed to optimize the use of the computational resources in such a multi-scale environment (Jeschke and Uhrmacher, 2008).

The present article aims at coupling a Brownian dynamics particle tracking method with the spatial Gillespie method to preserve the stochastic nature of the underlying signaling processes. Low-abundance species should for instance be always tracked as individual particles and high abundance species on the Gillespie level. Similarly subvolumes in focus of the simulation, e.g. receptor clustering on the plasma membrane, should be on the particle level. These two switches both on the species and the simulation volume level have to be accommodated in one simulation accordingly.

## 2 SYSTEM AND METHODS

We consider a reaction system consisting of *M* molecular species and *K* reaction channels with rate constants ***k*** = (*k*_1_,*...,k_K_*)*^T^*. The reactions take place in reaction compartment *Ω* (the cell or a sub-compartment thereof). In the following, we describe how the time evolution of this system can be modeled either on the particle or the population level as well as the relation between the levels.

On the particle level, we track the position ***x****_j_*(*t*) of the *j*^th^ molecule 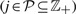 , where the particles follow Brownian dynamics. The species map *s*(*j*) = *i* indicates that the *j*^th^ molecule is of species *i*.

For a more coarse grained description, *Ω* can be subdivided into *U* subvolumes of volume *V*_1_,*...,V_U_*. We denote the number of particles in subvolume *ν* with ***N****^ν^* (*t*) = (*N*_1_*^ν^* (*t*),*..., N_M_^ν^* (*t*))*^T^*. Time evolution on this population level follows Markovian dynamics in our simulator.

For conversion from particle level to population level of the *i*^th^ species in *ν* we have to count all particles with corresponding properties: 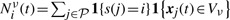 . Reversely, the underlying assumption of the population dynamics model is that the *N_i_^ν^* (*t*) molecules are uniformly distributed in *V_ν_*.

In our hybrid simulation, we denote for every volume *ν* which species is modeled in particle or population mode. Let us denote the subset of species tracked on the population level as ***Ñ^ν^*** (*t*). For every reaction *j* within *ν*, its waiting time *τ_j_^ν^* is distributed exponentially with parameter *a_j_*(***Ñ****^ν^* (*t*)), called the propensity of reaction *j* within *ν*. The waiting time *τ^ν^* for any reaction to occur in *ν* is exponentially distributed according to parameter *a*_0_(***Ñ****^ν^*) = ∑*_j_*_=1_*^K^ a_j_****Ñ****^ν^*). Starting from a given time *t*, the next event of reaction *j* in *ν* is according to Gillespie's algorithm (1976) at
(1)
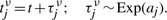

Diffusion of species *i* with diffusion coefficient *D_i_* into another volume *μ* is translated into a first-order transport reactions with propensity *a_i_*(***Ñ****^ν^*) = *k_i_^ν^*^→^*^μ^ N_i_^ν^*. The corresponding rate constant can be expressed as follows:
(2)
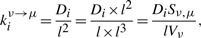

where *S_ν,μ_* is the surface/interface area of the cubic subvolumes. For convenience, we include these rates in ***k*** and thus *a*_0_(***Ñ****^ν^*). A transport reaction into *μ* at time *t*^′^ causes a state change ***Ñ****^μ^* (*t*) → ***Ñ****^μ^* (*t*^′^), which according to Gillespie's algorithm would require updating the precomputed waiting times *t_–_^μ^* in *μ*. Anderson (2007) proved that the remaining fraction of the time to the next reaction can simply be stretched according to the changed propensity
(3)
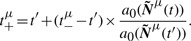


The remaining subset of ***N****^ν^* (*t*) is given by counting the individually tracked particles that currently reside in *ν*. Diffusion of the *j*^th^ particle is modeled according to Brownian dynamics as a random walk
(4)
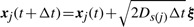

with ***ξ*** a three-dimensional zero mean Gaussian random variable with unit variance. In the present algorithm based on (Klann *et al.*, 2011a) the particles of radius *r_i_* are allowed to overlap because signaling molecules normally are of low abundance, so that these rare events can be omitted. Modeling of a physiologically crowded cytoplasm with non-overlapping molecules is computationally much more demanding (Ridgway *et al.*, 2008). Obviously steps into cellular structures (plasma membrane, nucleus, cytoskeleton, etc.) are rejected (Klann *et al.*, 2009). Particles can interact with each other if their distance is smaller than their collision radius *σ_ij_* = *r_i_* + *r_j_* [cf. Algorithm (1)], and a reaction based on the bimolecular rate constant *k_ij_* will be executed with probability (Lipková *et al.*, 2011)
(5)
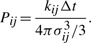


**Fig. 1.**
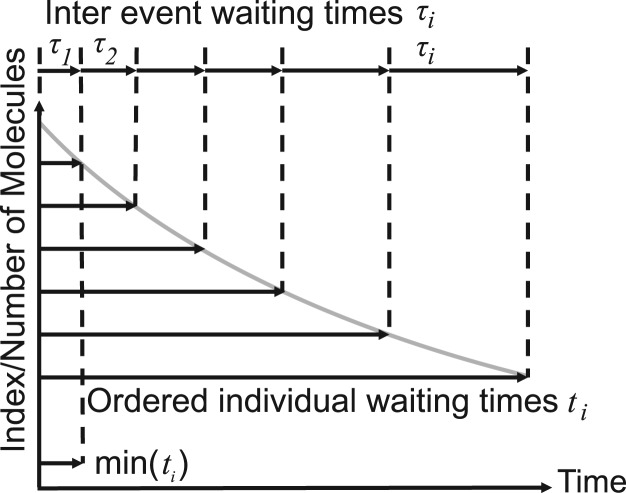
Equivalence of waiting times on the particle and population level (here assuming a first-order reaction like exponential decay)

The time *t_j_* to its first order reaction is calculated for each molecule individually when it is created based on Equation (1) with the single molecule propensity *a_j_*^1^ (*a_j_*^1^ = *k_j_*^1^ × 1, where *k_j_*^1^ is the sum of all first-order rate constants involving that species). The sequence of events of all molecules is then ordered, stored and updated as necessary Fig. 1, Arjunan and Tomita (2010); Byrne *et al.* (2010),. All first-order reactions up to current time *t* will be executed in that sequence (Klann *et al.*, 2011b). The actual reaction *i* that has to be executed out of the aggregate *k_j_*^1^ is found based on the probabilities *k_i_*/*k_j_*^1^.

Note the following equality: The minimum of the waiting time of *N* molecules, where each waiting time is calculated based on Exp(1), is Exp(*N*) (cf. Fig. 1). Thus, the individual method on the particle level and the global method on the population level are equivalent. On the population level, each of the identical *N_i_^ν^* molecules would be the one with minimal waiting time with probability 1/*N_i_^ν^*. More generally, suppose that the waiting time for *j*-th reaction, *τ_j_^ν^*, is distributed as Exp(*a_j_*), *j* = 1,*...,K*. Then the reaction *i* has the minimum waiting time with probability *a_i_*/*a*_0_. To see this observe that the required probability is given by

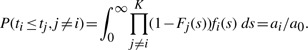

Here, *f_k_* and *F_k_*, respectively, denote the probability density function and the cumulative distribution function of Exp(*a_k_*).

## 3 ALGORITHM

With respect to coupling of Gillespie volume and individual particle tracking, we decided to partition the reaction set as follows:

1. First order reactions are stored on the species aggregation level, which is also used on the particle level for each species in each subvolume, i.e. one waiting time for each species. If no first-order reaction exists for species *i*, the waiting time *τ_j_^ν^* = ∞ (reaction times *t_j_^ν^*, *j* = 1,...,*M*).
2. Diffusion jumps are stored individually for each species in each subvolume (reaction times *t_j_^ν^*, *j* = *M* + 1,...,2*M*).
3. Higher order reactions are stored for each of the *R*_2_ higher order reactions in each subvolume (reaction times *t_j_^ν^*, *j* = 2*M* + 1,...,2*M* + *R*_2_).

These three groups match the behavior of the particle level, where first-order reactions are tracked along the molecules (of a species), diffusion jumps are triggered by the random walk process, and higher order reactions are triggered by (diffusion driven) collisions of molecules.

If we convert a subvolume from particle to Gillespie at *t*, all diffusion jump times and higher order reaction times will be initialized based on Equation (1). First-order reactions are treated differently, because each particle carries its next reaction time. The next reaction time of each species is therefore simply found by the minimum reaction time of all respective particles (cf. Fig. 1). The converted particles are then deleted.

The reverse conversion from Gillespie to particle creates *N_i_^ν^* particles uniformly distributed in the volume of the subvolume *ν* with individual properties inherited from the species properties. All predefined diffusion jump times and higher order reaction times are deleted because they will be triggered by the random walk of the molecules. The first-order reactions are, however, preserved in the following way:

- Arbitrarily, the first new particle of species *i* inherits the next reaction time *t_i_^ν^*. Note that all particles of one species are identical, so the term ‘first particle’ just refers to the memory location.
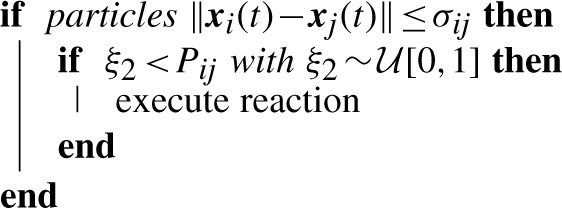
  **Algorithm 1:** Second-order (bimolecular) reactions on the particle level
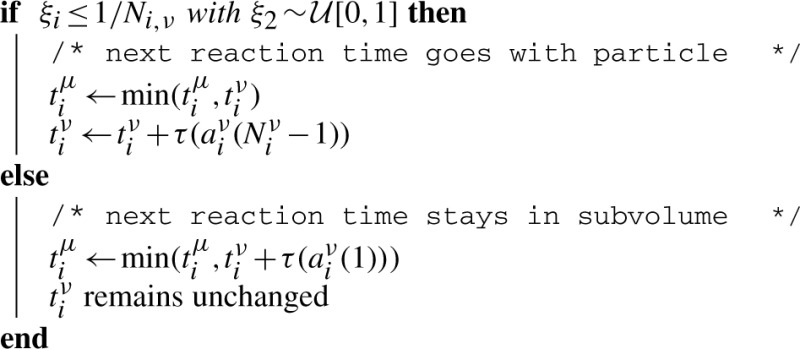
  **Algorithm 2:** Update scheme for the next first-order reaction after a jump from *ν* to *μ*, where the exponentially distributed waiting time is calculated by the indicated number of particles: either the remaining particles in *ν* or the one jumping from *ν* to *μ*
- All other particles of that species initialize their next reaction times as *t_j_* = *t_i_^ν^* + *τ*(*a_j_^ν)^* because their reaction has to be after the reaction of the first molecule accordingly.

The same approach is also followed for partial conversions of subvolumes, if not all but just a subset of the species is converted between the methods. All affected jump/higher order reaction waiting times on the Gillespie level are then updated based on Equation (3).

To be compatible with the above conversion approach, diffusion jumps also can carry the next first-order reaction time from subvolume *ν* to the new subvolume *μ*. The next time *t_i_^ν^* belongs to the jumping particle of species *i* with probability 1/*N_i_^ν^*. If a random number 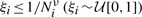, then the jumping particle is the next reacting particle. Algorithm 2 describes the executed process. The transferred time becomes the new time in the target volume *μ* if it is the minimum time. All affected jump/higher order reaction waiting times in both volumes on the Gillespie level are again updated based on Equation (3).

If particle *i* walks into a Gillespie level subvolume *μ*, then the next first-order reaction time there is likewise obtained by *t_i_^μ^* ← min(*t_i_^μ^*, *t_i_*). And if the diffusion jump out of Gillespie subvolume *ν* leads into a particle volume, then accordingly a particle is created which obtains the next reaction time *t_i_^ν^* with probability 1/*N_i_^ν^* and otherwise calculates its time as in Algorithm 1. However as the target subvolume is particle based, no global waiting time is tracked there.

Especially, if we have just one particle in the system, its initially assigned first-order reaction time will be preserved throughout the simulation. This approach also ensures that frequent switching between particle and Gillespie volume tracking do not require too many re-initializations of the waiting times. Otherwise frequent switching could accumulate to a substantial error in the reaction rate if the switching intervals are in the range of the waiting times.

Thus, the suggested tracking and updating method on the Gillespie level ensures the compatibility with the particle tracking level but is not necessarily optimal with respect to its own performance due to the large number of stored reaction times and the time necessary to access and order them. As the Gillespie level still integrates time much faster than the particle level, this performance loss is, however, not relevant for the overall performance of the simulation.

Eventually also bimolecular reactions across the levels might occur in the simulation: let us assume that *N_j_^ν^* molecules of species *j* are in the Gillespie subvolume with volume *V_ν_* and we have a bimolecular reaction with rate constant *k_ij_* with species *i*, which is tracked on the particle level. Concentration-based mass action kinetics requires that the reaction rate is *k_ij_c_j_c_i_* = (*k_ij_N_j_^ν^* / *V_ν_*)*c_i_*, so the reaction can be treated as a first-order reaction with rate constant *k_i,ν_* = *k_ij_N_j_^ν^* / *V_ν_*. As this rate ‘constant’ depends on the time varying *N_j_^ν^*, we simulate these quasi first-order reactions in the classical particle-based scheme [cf. Andrews and Bray (2004); Klann *et al.* (2011a)]: each molecule *i* in the volume *ν* reacts with probability
(6)
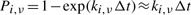

in every timestep. This means, that a random number 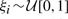 is compared with *P_i,ν_* for all molecules in every time step. This scheme also resembles the scheme of bimolecular reactions, where the probability is given by Equation (5), and Klann *et al.* (2011b) have extended it for dependent bimolecular reactions in a scaffold accordingly.


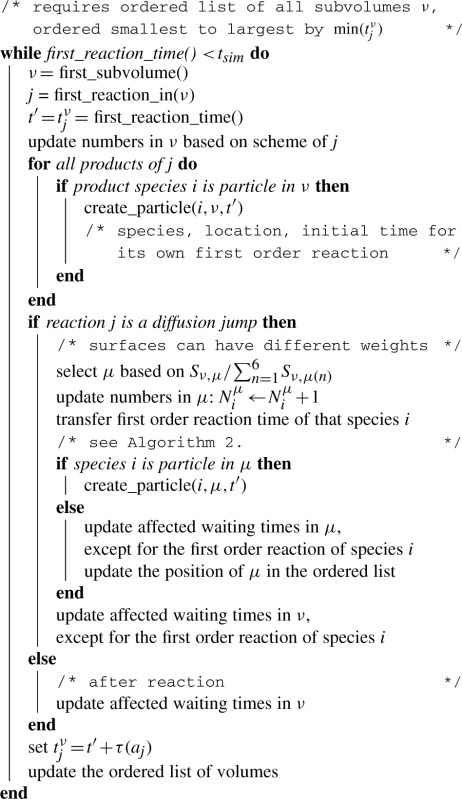


**Algorithm 3:** Algorithm of the Gillespie block. Note that particle conversions into Gillespie also change *N^ν^*, which requires to update reaction times and the position of the volume in the ordered list. The exponentially distributed waiting time can be simply computed as *τ*(*a_j_*) = 1/*a_j_* log(1/*ξ*) using 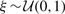


Algorithm 4 gives an overview of the complete simulation. Note that the steps (1)–(4) could be executed in any order. Visualization of the particles/volumes was done with *BioInspire* from ScienceVisuals, Lausanne, Switzerland (de Heras Ciechomski *et al.*, 2008) and POVray (www.povray.org).

## 4 IMPLEMENTATION AND PERFORMANCE

The method is implemented in Fortran, integrated into the functionality of the previously published particle-based methods (Klann *et al.*, 2011a, b, 2012). This includes first order (*A* → ...) and second order (*A* + *B* → ...) mass action kinetics reactions, Michaelis–Menten enzyme kinetics (*E* + *S* → *E* +...) and binding and dissociation to/from plasma membrane, cytoskeleton and the nucleus. In particle mode, molecules can diffuse along the plasma membrane, the nucleus, through the cytoplasm or walk along the cytoskeleton. So far the simulation is not parallelized, but a parallel version in C/C++ is in preparation.

**Fig. 2.**
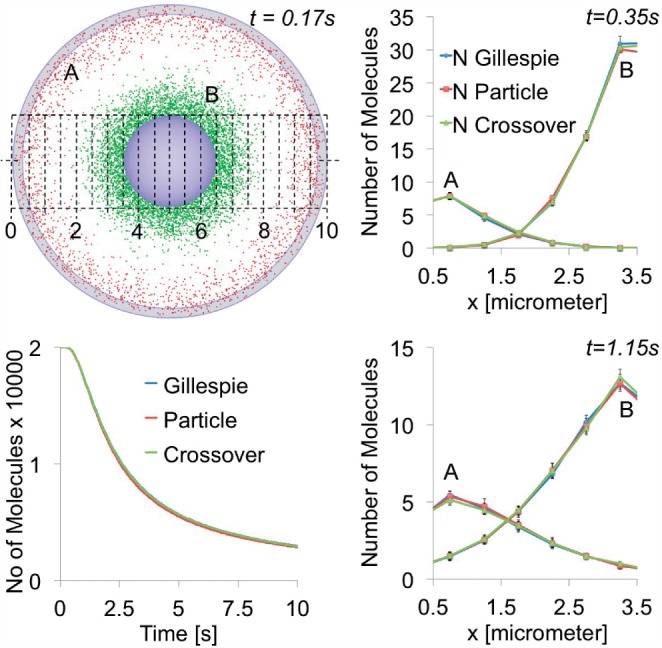
Testcase with *A* + *B* → 0, where *A* starts from the plasma membrane and *B* from the nucleus. The grid shows the discretization in x direction used for evaluation of the spatial distribution

Here, we show the testcase *A* + *B* → 0, where *A* starts from the plasma membrane and *B* from the nucleus with 20 000 molecules each in Figure 2. We run the simulation with both species in particle mode, both in the population-based Gillespie mode, and A as particle, whereas B is in Gillespie mode. Figure 2 shows no differences between the simulations however, the larger figure in the supplementary material shows that the particle-based simulation leads to slightly faster reaction rates in the initial mixing phase around 1.5 s. This could be an effect of local fluctuations that do not cancel out due to the nonlinearity and nonstationarity of the mixing process. More test cases and performance numbers are presented in the supplementary material, testing the different reaction schemes and combinations of the particle- and Gillespie-based molecules. Further performance numbers (for the system discussed in the next section) are given in Table 1.

**Table 1.** Performance of the hybrid simulation for the model system

MAPKpp	Phosphatase	Volumes (*U*)	*T_cpu_*
Always particle	Always particle	5592 (*L* = 20)	174 min
Always particle	Always Gillespie	872 (*L* = 10)	94 min
Always particle	N/A ^a^	5592 (*L* = 20)	41 min
Particle at membranes	Always Gillespie	17 488 (*L* = 30)	49 min
Particle at membranes	Always Gillespie	5592 (*L* = 20)	13 min
Particle at membranes	Always Gillespie	872 (*L* = 10)	15min^b^

Tested on a Win 7 Pro Intel i7 2600K at 3.5 GHz, 8GB RAM.

^a^Dephosphorylation is modeled by a first-order reaction with *k*_p_^′^ = 1.0 s^−1^ instead, which is the effective rate of the bimolecular reaction. This shows that the pair finding of the bimolecular reaction dominates *T_CPU_*.

^b^Because of the more coarse grained grid, a bigger volume fraction along the plasma membrane was in particle mode, compared with the settings with more subvolumes.


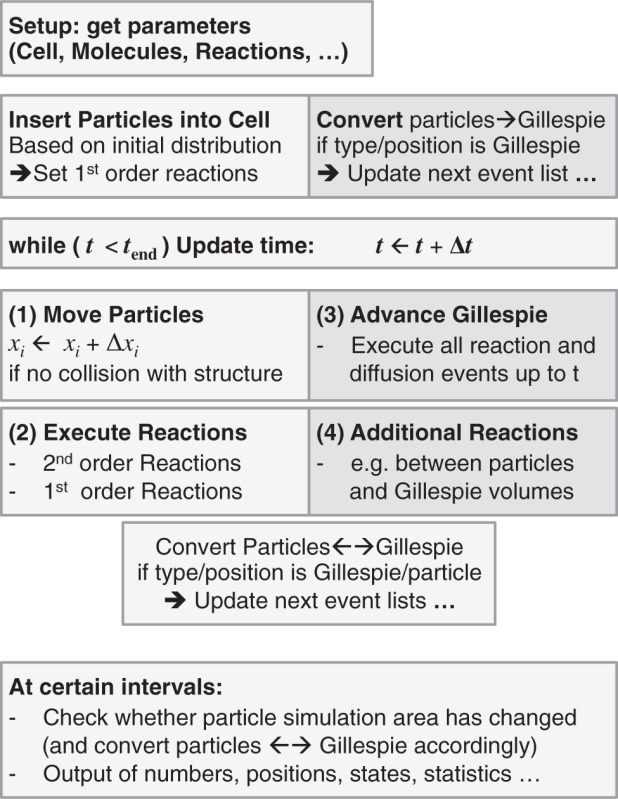


**Algorithm 4:** Overview of the simulation steps

## 5 DISCUSSION

Spatial and temporal effects can play a major role in signal transduction (Kholodenko *et al.*, 2010). This holds especially for the transport of active signaling molecules from the plasma membrane where the signal is detected by receptors toward the nucleus, where it has to trigger gene activation. A common motive in signal transduction is the mitogen-ctivated protein kinase (MAPK) cascade, where the receptor and upstream components cluster together at the plasma membrane, and MAPK enters the nucleus. The active, phosphorylated form of MAPKpp is constantly deactivated in the cytoplasm. The fraction of active molecules reaching the nucleus, therefore, depends on the relationship between the speed of transport (diffusion) and the speed of the deactivation (reaction) (Kholodenko, 2006).

**Fig. 3.**
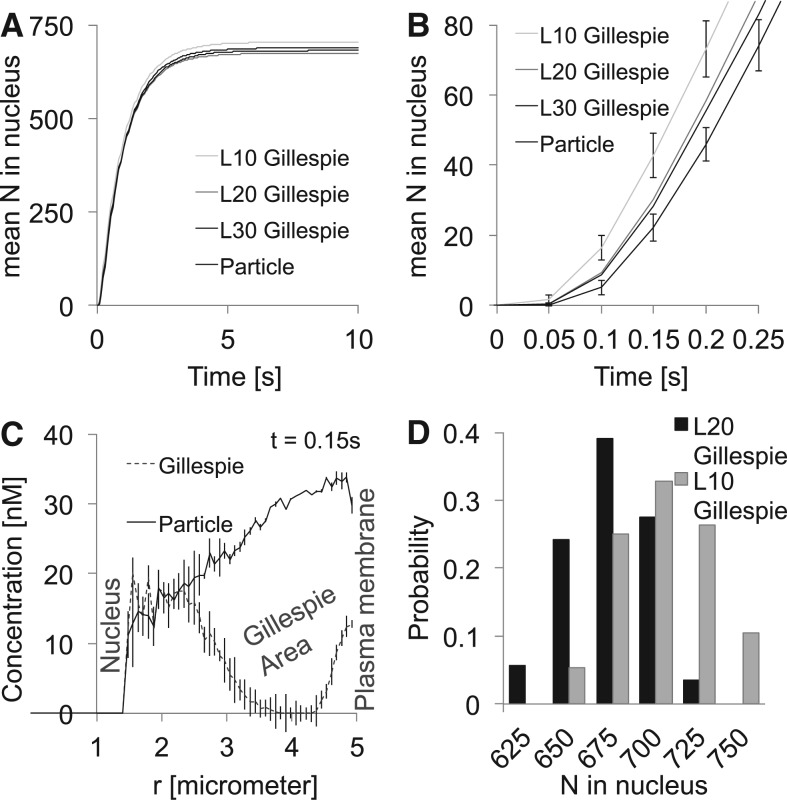
(**A**) Mean of the MAPKpp molecules that reached the nucleus. **(B)** Initial phase of **(A)**. **(C)** Spatial distribution of the MAPKpp molecules—counting only the particle molecules (the conversion from cubic areas to the spherical symetric radial distribution hides the sharp cutoff from the particle to the Gillespie area). **(D)** Distribution of the number of MAPKpp molecules that reached the nucleus within 10s

We simulated this process in a spherical cell with diameter 10*μ*m with a spherical nucleus (diameter 3*μ*m, located in the cell center) A total of 10 000 MAPKpp molecules start initially from the plasma membrane with D_MAPK_ = 10*μ*m^2^/s and can enter the nucleus with a rate constant of 1.8*μ*m/s (given the ‘concentration’ of the nuclear membrane in the cytoplasm, this leads to an effective nuclear import rate constant of 0.1s^−1^). A total of 30 577 phosphatase molecules ([P] = 1 × 10^−7^ M) dephosphorylated MAPKpp→MAPK with *k*_p_ = 1 ×10^7^ M^−1^ s^−1^. We simulated this process for 10s with Δ*t* = 2.6 × 10^−5^s for various combinations and discretization levels (*L* = 10, 20 or 30 subvolumes along the cell diameter) of the hybrid particle and Gillespie method (cf. Table 1). The results are shown in Figure 3. Depending on the size/number of the Gillespie subvolumes, the runtime could be reduced by more than 90%. However, large subvolumes propagate the molecules slightly faster in the initial phase (Fig. 3B). The improved performance of the hybrid simulation allows us to compute the distribution of the fraction of active signaling molecules that entered the nucleus (Fig. 3D). The discretization error is also visible in the distribution, which is shifted to higher particle numbers for lower *L*. The area close to the nucleus was always modeled in particle mode. Figure 3C shows that also the hybrid Gillespie simulation leads to the correct concentration there.

**Fig. 4.**
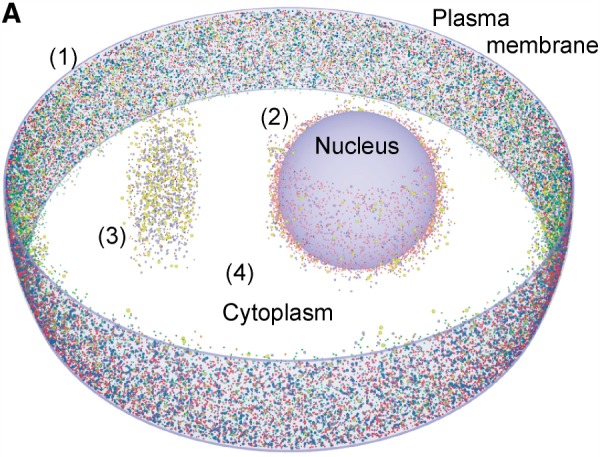
Visualization of a slice of a cell with particle tracking along (1) plasma membrane and (2) nucleus and (3) in an extra section of the cytoplasm, while (4) the remaining cytoplasm is simulated in Gillespie mode (rendered with POVray)

With respect to other works, analyzing the importance of receptor clustering and the spatial organization at the membrane for signaling (Costa *et al.*, 2009; Geier *et al.*, 2011; Mugler *et al.*, 2012), our method allows to focus the computational resources to the membrane, whereas still the complete cell is tracked in 3D (Fig. 4). similarly, our method allows including membrane trafficking and recept-mediated endocytosis (Klann *et al.*, 2012).

In the following, we want to analyze how far molecular crowding can be modeled on the Gillespie level. This was also investigated by Nicolau and Burrage (2008), Jeschke and Uhrmacher (2008) and Lampoudi *et al.* (2009). Let *ϕ* denote the free volume fraction of the cytoplasm (i.e. the fraction which is accessible to the molecules of interest). Crowding leads to *ϕ* < 1 and the reactants in the remaining volume *V_ν_*^′^ = *V_ν_ϕ* have an effectively higher concentration, such that they react in bimolecular mass action kinetics with a faster rate (Klann *et al.*, 2011a; Lampoudi *et al.*, 2009). This effect is correctly included in our simulation, because bimolecular rate constants given in [M^−1^s^−1^] have to be divided by *V_ν_*^′^ anyway for the Gillespie level (and an additional factor to convert from the liter in M = mol/l to the used length scale and single particles). For substrates *h* and *i* and length in *μ*m

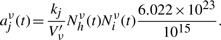


**Fig. 5.**
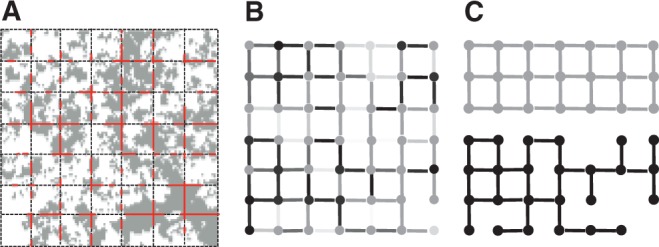
**A** Obstacles in the cell (from Transmission electron microscopy image) and simulation grid. **B** Resulting graph/network of the diffusion system, where the node and edge weight corresponds to the local free volume fraction. Note that the diffusion rate constant is given as the fraction of edge/source node weights, such that it can be different in the two directions along an edge. **C** Limit cases depending on the chosen discretization: all nodes/edges have the same (average) weight, e.g. if *l* is much larger than the length scale of the obstacles, or the resulting network becomes binary with weights 1 or 0 respectively if the obstacles have the same size like *l*

Diffusion, in contrast, is reduced by crowding. The jump rate constant on the Gillespie level [Equation (2)] likewise contains the properties of the volume. If the size of the obstacles becomes much smaller than *l*, such that the objects are uniformly/homogeneously distributed in the volume, however, both *V_ν_* and *S_ν,mu_* will be scaled by the same *ϕ*. Thus, the effect cancels in Equation (2). Only if we go to a fine discretization of space, such that *S_ν,mu_* becomes a random number with mean *l*^2^*ϕ*, then the crowding effect becomes visible in the resulting diffusion (cf. Fig. 5). These networks and the resulting diffusion can also be analyzed with percolation clusters (Gefen *et al.*, 1983).

**Fig. 6.**
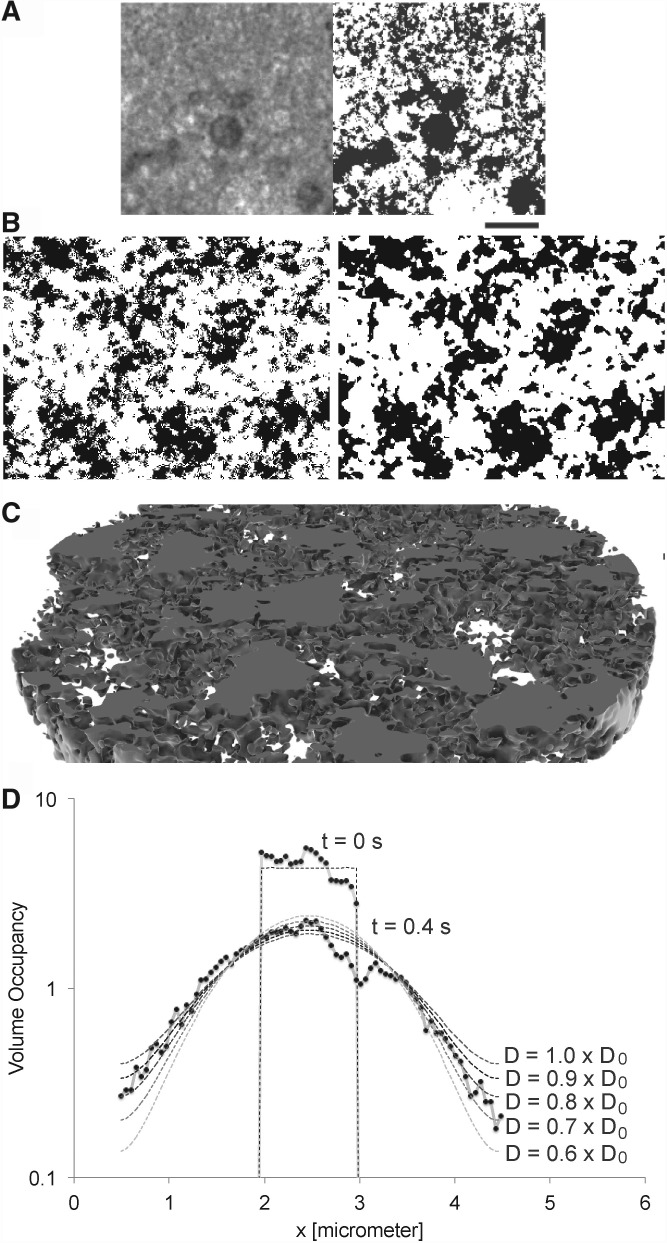
**A** Based on the statistics of binarized Transmission electron microscopy (TEM) images from (Hiroi *et al.*, 2011) **B** realistically looking intracellular obstacle geometries were generated (Hiroi *et al.*, 2012). **C** shows a 3D section of a generated volume, which looks similar like other 3D observations of membrane enclosed compartments like e.g. ER or Golgi (rendered with BioInspire from ScienceVisuals). **D** Diffusion through such a structured volume, simulated on the Gillespie level. Apparently at *x* = 3*μ*m a high obstacle density leads to a lower particle density

We tested this in an intracellular environment as shown in Figure 6, where the total diameter of 4.928*μ*m was divided into 94 subvolumes in each direction (4 million subvolumes in the sphere, requiring 1 GB of memory in the simulation). At this scale [*l* = 52nm, which is 3 pixel of the original binarized transmission electron microscopy (TEM) image of Hiroi *et al.* (2011)] we see a reduction in diffusion (cf. Fig. 6D). Still, the diffusion is only reduced to *D*_eff_ = 0.9*D*_0_, whereas with particle tracking, we find *D*_eff_ = 0.7*D*_0_ (Hiroi *et al.*, 2012). The strong deviations in Figure 6D arise from the locally varying *V_ν_*, apparently at *x* = 3*μ*m a big object reduces the available volume. This result underlines the fact that the reduced diffusion originates from the tortuosity of the structured volume: the particles do not move slower but have to go longer ways around the obstacles, which delays their arrival (Klann *et al.*, 2011a).

Finally, it should be noted that reactions can be diffusion controlled (Morelli and Ten Wolde, 2008; Rice, 1985). Molecules cannot react faster than *k_D_* = 4*π σ D* (where *D* is the sum of both diffusion coefficients). If *k_j_* is close to *k_D_*, the observed rate is determined by the rate of collisions. Thus, if the subvolumes of the Gillespie method are too small, the method will not find enough pairs and underestimate the true reaction rate (Gillespie, 2009). Gillespie (2009) calculated the necessary correction factor.

In this low concentration and diffusion-controlled regime, particle-based methods still give correct results (Klann *et al.*, 2011a; Morelli and Ten Wolde, 2008). Therefore, we are planning to introduce a controller which switches from Gillespie to particle if the local concentration falls below the threshold. Note that particle tracking also is not expensive if just a few molecules have to be tracked, whereas the mean jump frequency grows with 1/*l* [cf. Equation (2)], making high-resolution spatial Gillespie simulations even more expensive.

In addition, we will develop a user interface to allow a better control of the simulation. This will also include interactive visualization of the molecules in the simulation (de Heras Ciechomski *et al.*, 2008; Falk *et al.*, 2010).
